# Spectral and binaural loudness summation of equally loud narrowband signals in single-sided-deafness and bilateral cochlear implant users

**DOI:** 10.3389/fnins.2022.931748

**Published:** 2022-08-22

**Authors:** Hongmei Hu, Laura Hartog, Birger Kollmeier, Stephan D. Ewert

**Affiliations:** ^1^Medizinische Physik and Cluster of Excellence “Hearing4all”, Department of Medical Physics and Acoustics, Universität Oldenburg, Oldenburg, Germany; ^2^Hörzentrum Oldenburg gGmbH, Oldenburg, Germany

**Keywords:** spectral loudness summation, binaural loudness summation, categorical loudness, single-sided-deafness, cochlear implant, bilateral cochlear implant fitting

## Abstract

Recent studies on loudness perception of binaural broadband signals in hearing impaired listeners found large individual differences, suggesting the use of such signals in hearing aid fitting. Likewise, clinical cochlear implant (CI) fitting with narrowband/single-electrode signals might cause suboptimal loudness perception in bilateral and bimodal CI listeners. Here spectral and binaural loudness summation in normal hearing (NH) listeners, bilateral CI (biCI) users, and unilateral CI (uCI) users with normal hearing in the unaided ear was investigated to assess the relevance of binaural/bilateral fitting in CI users. To compare the three groups, categorical loudness scaling was performed for an equal categorical loudness noise (ECLN) consisting of the sum of six spectrally separated third-octave noises at equal loudness. The acoustical ECLN procedure was adapted to an equivalent procedure in the electrical domain using direct stimulation. To ensure the same broadband loudness in binaural measurements with simultaneous electrical and acoustical stimulation, a modified binaural ECLN was introduced and cross validated with self-adjusted loudness in a loudness balancing experiment. Results showed a higher (spectral) loudness summation of the six equally loud narrowband signals in the ECLN in CI compared to NH. Binaural loudness summation was found for all three listener groups (NH, uCI, and biCI). No increased binaural loudness summation could be found for the current uCI and biCI listeners compared to the NH group. In uCI loudness balancing between narrowband signals and single electrodes did not automatically result in a balanced loudness perception across ears, emphasizing the importance of binaural/bilateral fitting.

## Introduction

In normal hearing (NH) listeners, the binaural system is important for providing cues to segregate target signals from competing (or interfering) sounds (Cherry, 1953) and to identify the location of sound sources (Blauert, 1997). In order to partially recover the benefit of binaural hearing, the number of bilateral cochlear implant (biCI) users as well as unilateral CI (uCI) users with normal hearing in the unaided ear (single-sided deafness) increased in recent years (Zeng et al., 2008; Peters et al., 2010; Arndt et al., 2011; van Zon et al., 2015; Wouters et al., 2015). However, a high variability in the binaural performance of cochlear implant (CI) users is reported in the literature (Litovsky et al., 2012; Dorman et al., 2014; Steel et al., 2015). This variability might be related to, but not limited to, current CI coding techniques (e.g., Hu et al., 2018), bilateral electrode mismatching (e.g., Kan et al., 2013; Hu and Dietz, 2015), and bilateral aspects of loudness-based fitting of the electrical dynamic range (e.g., Goupell et al., 2013; Fitzgerald et al., 2015; Hu and Dietz, 2015). In the clinic, fitting is normally performed in order to obtain the so called ‘Map’ (electrical parameters) stored in the speech processor for optimizing the performance of each individual CI. Besides impedance measurements of the electrodes and the deactivation of electrodes for example due to short cuts, the main component of the first fitting are loudness-related measurements: the hearing threshold level (T-level), maximum comfortable level (M-level) to determine the electrical dynamic range (DR), and loudness balancing tasks between the single electrodes (Vaerenberg et al., 2014). Such ‘Map’ derived from single-electrode stimuli does not take into account the temporal and spatial patterns of the speech processor output (McKay et al., 2001). In practice, depending on the recipient’s perception, the audiologist can perform global or channel adjustments of the T- and M-levels (Wolfe and Schafer, 2014). Regarding bilateral fitting, CI programming software of different manufactures typically provides a binaural mapping, however, no standardized procedure of the bilateral CI fitting exists (e.g., Goupell et al., 2013). Clinicians often adjust levels for bilateral loudness balance by adjusting overall levels for broadband stimuli, such as their voice. In general, loudness perception of such broadband, binaural stimuli is highly relevant in daily life (e.g., Oetting et al., 2014). For such stimuli, loudness perception involves effects of spectral loudness summation (increasing loudness with increasing spectral bandwidth; Zwicker et al., 1957; Florentine et al., 2011) as well as binaural loudness summation (increased binaural loudness perception compared to the monaural presentation; Moore and Glasberg, 2007; Florentine et al., 2011; Moore et al., 2014, 2016).

Regarding binaural loudness perception and fitting in hearing impaired (HI) listeners with conventional acoustic hearing aids, recent studies (Oetting et al., 2016; van Beurden et al., 2018, 2020) found a large variability of binaural broadband loudness perception in HI listeners after restoring the individual loudness perception of monaural narrowband signals due to frequency-dependent amplification. Ewert and Oetting (2018) introduced a loudness-based measurement procedure using a combination of narrowband sounds at equal loudness, enabling a direct comparison of loudness perception in NH and aided HI listeners. Their results showed an increase of binaural broadband loudness in HI compared to NH listeners, suggesting to include binaural broadband signals in hearing aid fitting procedures (e.g., Ewert and Oetting, 2018). The origin of such an increased binaural loudness is not yet clear and higher stages of the auditory pathway are likely involved. Thus it is conceivable that such an effect might also occur in bilateral or bimodal CI users. Given that a similar and balanced loudness perception in both ears can be assumed to support a more natural sound impression and might be beneficial for binaural fusion in biCI as well as uCI, where the acoustic sound and the electrical stimulation have to be combined to common percept, similar binaural fitting procedures as suggested for HI might also be beneficial for CI users.

Regarding spectral and binaural loudness summation in CI users, basic research was in the focus so far. McKay et al. (2001) investigated the effect of electrode separation, stimulation rate, and level on dual-electrode loudness summation using a loudness-balancing procedure. The level of the dual-electrode stimulus was reduced in all conditions to match the loudness of the single-electrode stimulus. Different electrode separations, three stimulation rates (250, 500, and 1000 pps), and two levels (comfortably loud level and 50% DR) were tested. They found the effect of electrode separation to be smaller than the effect of level and stimulation rate. For the assessment of loudness perception in CI listeners, Theelen-van den Hoek et al. (2014) showed a good reliability of categorical loudness scaling using direct stimulation of the CI electrodes in the electrical domain comparable to the acoustical domain with adaptive categorical loudness scaling (ACALOS; Brand and Hohmann, 2002; ISO16832, 2006). Theelen-van den Hoek et al. (2015) investigated the spectral loudness summation for electrical stimulation by calculating the level differences that produced equal loudness between single-electrode (basal or apical) stimuli and multi-electrode stimuli (2 or 4 electrodes with the same overall stimulation rates corresponding to the single-electrode stimulation rate). They found a significant amount of spectral loudness summation in a subset of the CI users and that the spectral loudness summation has more effect on the perceived loudness than the stimulation rate with increasing categorical loudness. Bilateral loudness perception using single-electrode stimuli for two CI users was investigated in Hoesel and Clark (1997). They reported that bilateral stimulation was judged on average two times louder in comparison with unilateral presentation. These findings were verified for broadband signals by van Hoesel (2004) using broadband pink-noise bursts presented via audio-input connector to the listeners’ CIs. Their results also showed a two-fold increase of loudness of the bilateral stimulation compared to the unilateral stimulation in a large part of the dynamic range. Kordus and Żera (2017) tested 15 biCI users via their own processors, and found a large variability of bilateral loudness perception, similar to the large variability observed in HI listeners (Oetting et al., 2016; van Beurden et al., 2018). Blamey et al. (2000) measured binaural loudness perception in unilateral CI users with a residual hearing in the contralateral ear aided with a hearing aid. Their results showed that the binaural presentation was on average judged with higher categories than the monaural presentation with equal loudness.

Taken together, based on recent results for HI listeners, binaural loudness summation for (everyday life) broadband stimuli appears relevant for CI fitting, considering aspects of pleasant loudness perception as well as fusion of the auditory image across ears. Particularly for unilateral CI listeners with acoustic hearing in one ear, methods to compare loudness perception for simultaneous electric and acoustic stimulation are required.

The primary goal of the current study was to investigate spectral and binaural loudness summation in biCI and uCI users compared to NH listeners in order to assess the importance of bilateral broadband loudness fitting for biCI and uCI users. Therefore, binaural measurements with simultaneous stimulation in the acoustical and electrical domain are required. The secondary goal was to develop suggestions for a bilateral loudness fitting procedure within a common framework for electric, acoustic, and electric/acoustic stimulation. For this, the equal categorical loudness noise (ECLN) procedure (Ewert and Oetting, 2018) based on an Adaptive CAtegorical LOudness Scaling (ACALOS; Brand and Hohmann, 2002; ISO16832, 2006) was adapted to an equivalent electrical procedure. Direct stimulation of the CI electrodes in biCI users and simultaneous acoustical and electrical stimulation was used in uCI users to enable binaural measurements. The ECLN procedure offers an advantage particularly for binaural measurements with uCI users, given that sound intensity classically used in the acoustical domain and electrical charges used in the electrical domain are not directly comparable. Using ECLN, loudness serves as common (perceptual) parameter across both ears, independent of the different modes of stimulation. Another advantage of the ECLN procedure is that it allows a direct comparison of (binaural) loudness perception in CI users and in the group of HI listeners in Ewert and Oetting (2018).

Two different binaural ECLN procedures were tested in the acoustic, electric, and combined acoustic and electric domain in three groups of NH, biCI, and uCI listeners: an original binaural ECLN as in Ewert and Oetting (2018) and a proposed modified binaural ECLN (modECLN) for coping with potentially different spectral loudness summation in two ears, where binaural loudness perception can be dominated by the loudness in a single (e.g., either electric or acoustic) ear. Here, the proposed binaural modECLN measurement was based on the monaural ECLN results, designed to provide the same broadband loudness in both ears. In order to cross validate the proposed binaural modECLN procedure, a loudness balancing experiment was additionally performed to compare the self-adjusted narrowband loudness differences (between the left and right ear) with those assumed in the suggested modECLN (based on comparable monaural loudness perception in both ears).

## Materials and methods

### Listeners

The NH group consisted of eight listeners (four female and four male), aged 21 – 33 years (mean 29.5 years). All NH listeners had pure-tone thresholds ≤ 20 dBHL in the audiometric frequencies from 125 to 8000 Hz and the pure-tone average over 500, 1000, 2000, and 4000 Hz (PTA4) was between 0 and 10 dBHL. Four post-lingually uCI users and four biCI users (one listener pre-lingually deafened) participated in this study. All CI listeners use MED-EL implant systems and had at least 1 year CI experience. Information about the biCI and uCI listeners and the involved probe electrodes is summarized in Table 1. All listeners received an hourly compensation including traveling expenses for the CI listeners.

**TABLE 1:** Demographic information of the CI participants including gender, age at testing, etiology, years of electric experience for the left and right implant (yrs exp CIs), the test electrodes, and PTA4 of non-implanted ear.

ID	Sex	Age	Etiology	Yrs exp CIs L/R	Test electrodes	PTA4 of non-implanted ear
uCI1	F	21	Unknown	3/–	2 4 6 8 10 11	0
uCI2	M	68	Sudden hearing loss	–/4	2 5 6 9 11 12	13.75
uCI3	M	54	Sudden hearing loss	2.5/–	2 4 6 8 10 11	6.25
uCI4	M	54	Unknown	1/–	2 4 6 8 10 11*	7.5
biCI1	M	57	Congenital hearing loss	3/4**	2 3 5 7 9 10/2 4 6 8 10 11	–
biCI2	M	75	Unknown	4/9	2 4 6 8 10 11/2 3 5 8 10 11	–
biCI3	M	24	Congenital hearing loss	14/10	2 4 6 8 10 11/3 4 6 8 10 11	–
biCI4	M	74	Processing hearing loss	4/2	2 4 6 8 10 11/2 4 6 8 10 11	–

*Standard frequency map for 12 active electrodes was used; ** re-implantation after 2 years.

### Stimuli

Six narrowband third-octave low-noise noises (Kohlrausch et al., 1997) with center frequencies of 250, 500, 1000, 2000, 4000, and 6000 Hz were used for loudness scaling in the acoustical domain. Each stimulus had duration of 1 s including 50 ms onset and offset Hanning ramps, respectively.

The electrical stimuli were monopolar biphasic pulse trains with a rate of 900 pulses per second (pps). The stimulation rate of 900 pps per channel enables comparison with previous loudness perception investigations (e.g., Theelen-van den Hoek et al., 2014, 2015; Busby and Au, 2017). The interphase gap was 2.1 μs and the default pulse width was set to 50 μs. The pulse width was adjusted (with a step size of 10 μs) for some listeners during the pretests in order to stay below the compliance limit of the CI and to enable all required presentation levels for the loudness scaling (see Table 2). To transform the acoustical stimuli to the electrical domain, the Hilbert envelope *H*(*n*) of each narrowband signal was extracted to modulate the pulse trains. This resulted in six amplitude modulated pulse trains (normalized to obtain a maximum amplitude of one). These pulse trains were mapped to the corresponding electrode’s DR according to individual hearing threshold level (T-level) and maximum comfortable level (M-level), between 0 and 1200 current levels (CL) of MED-EL, where 1 CL corresponds approximately to 1 μA. Motivated by the use of discrete pulse trains and the limitation of the maximum to 1200 CL, the crest factor of the envelope was reduced to $\tilde{H}\,\left(n\right)$before sampling using

**TABLE 2:** Used pulse width in μs and measured dynamic range (DR) in CL for the six used electrodes corresponding to 0.25, 0.5, 1, 2, 4, 6 kHz.

ID	Pulse width (us)	DR (CL)					
		0.25 kHz	0.5 kHz	1 kHz	2 kHz	4 kHz	6 kHz
uCI1	60	750	722	961	814	797	608
uCI2	50	667	488	790	922	858	669
uCI3	50	453	538	600	497	547	430
uCI4	80	663	732	798	685	549	435
biCI1(L/R)	100	477/329	650/411	630/559	452/399	191/317	100/283
biCI2(L/R)	40	335/264	526/471	503/498	337/392	230/294	199/429
biCI3(L/R)	50	442/295	423/318	613/387	600/308	351/305	438/307
biCI4(L/R)	50	360/315	539/394	604/542	510/535	296/341	276/319


$$\tilde{H}\,\left(n\right)=a\,H\,\left(n\right)+\left(1-a\right)\,\bar{H\,\left(n\right)}$$

with *a* = 0.5 and the mean of Hilbert envelope $\bar{H\,\left(n\right)\,}$of the narrowband noise stimuli.

The ECLN (Ewert and Oetting, 2018) and the here suggested modECLN consisted of the sum of the six narrowband noises at equal loudness for each individual noise. In the CI, this corresponds to the sum of the six electrical (single-electrode) stimuli at equal loudness. The clinical frequency map of the listeners was used to identify the six electrodes best corresponding to the six center frequencies of the noises. These probe electrodes were then used for stimulation (see Table 1). Analog to the a basic continuous interleaved sampling (CIS) strategy (Wilson et al., 1991) used in Hu et al. (2018), the electrodes were stimulated sequentially from apical to basal.

### Apparatus

The experimental setup consisted of an acoustical and an electrical stimulation. For the acoustical stimulation, the stimulus was DA-converted in the audio interface (RME Fireface UCX) and then amplified (Tucker-Davis HB7) before it was played back over headphones (Sennheiser HDA 200). For the electrical stimulation, pulse trains were directly delivered to the CIs using a MED-EL research interface box (RIB II, University of Innsbruck, Austria) which communicated directly with implants via a National Instruments I/O card, optical isolation interface box, and two telemetry coils, bypassing any speech processor. A graphical user interface (GUI) modified from Hu et al. (2014) and Hu and Dietz (2015) was used to input the listener’s information (e.g., implant type and implant ID) and experiment parameters (e.g., test electrode, pulse parameters), to execute the hearing tests (e.g., hearing threshold, ACALOS), and to control the electrical stimulation via the stimulation computer. The system enables acoustical or electrical stimulation as well as a simultaneous combination of both. All the psychoacoustic experiments and the ACALOS procedure were implemented in the freely available AFC framework for MATLAB, a psychophysical-measurement package (Ewert, 2013).

All experiments took place in a sound-proof booth. During the measurement procedure, the listeners were instructed to enter their response via a touchscreen or a computer mouse. For headphone calibration an artificial ear (type 4153, Bruel and Kjaer) with a 0.5-inch microphone (type 4192, Bruel and Kjaer), a microphone preamplifier (type 2669, Bruel and Kjaer) and a measuring amplifier (type 2610, Bruel and Kjaer) were used. As implemented in the ACALOS toolbox of the AFC framework, the free-field equalization according to ISO389-8 (2004) was applied for signal calibration. The electrical stimuli were verified using one or two detector boxes (the MED-EL CI simulators) and an oscilloscope. Furthermore, there was no presentation as well as perceived time delay between the acoustical and electrical stimulation, which was checked with both an oscilloscope and informal listening tests of an uCI listener.

### Test procedure

**FIGURE 1:**
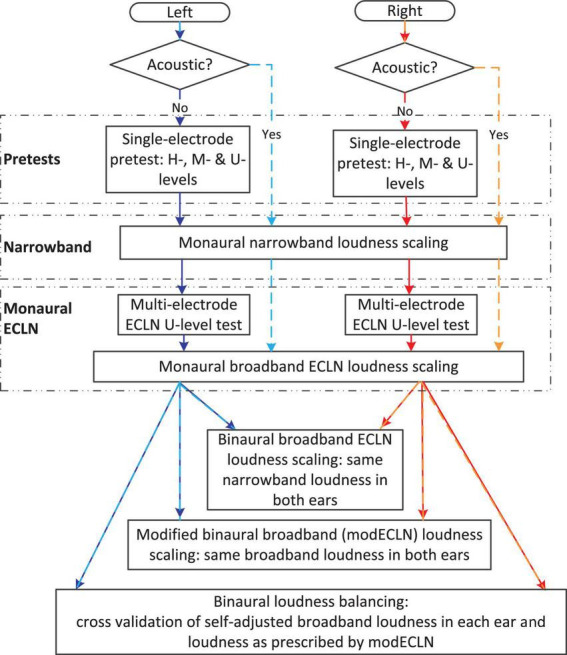
Schematic overview of the test procedures.

The measurements consisted of two sessions, each lasting about 2 hours. After pretests in the first session, three different loudness perception experiments were performed in the first and second session to compare the amount of loudness summation in the three listener groups: the monaural narrowband noise measurements, the monaural and binaural broadband ECLN measurement, and the binaural broadband modECLN measurement. In addition, a self-adjusted binaural loudness balancing experiment was performed to cross check whether the proposed modECLN produces equally loud broadband signals in the left and right ear. Figure 1 shows the schematic overview of the test procedures.

#### Pretests in the electrical domain

To determine the electrical DR and the maximal presentation level for the ACALOS procedure in the electrical domain, three different pretests were conducted using the same self-adjusted procedure, based on the pretest procedure used in Hu et al. (2014) and Hu and Dietz (2015). The listeners were asked to adjust the presentation level of all electrical narrowband (single-electrode) stimuli to the just audible level (T-level), the maximum comfortable level for a longer presentation duration (M-level), and additionally to an uncomfortable but maximum tolerable presentation level (U-level), by pressing either the “softer” or “louder” button on the touch screen. The use of the additional U-level aimed to obtain more judgments in the higher loudness range. The presentation level was adjusted between 0 and 1200 CL until the listener finished the task by pressing the “done” button. In order to familiarize the listeners with the task and to determine the range of the T- and M-level, the procedure was tested twice for the electrode corresponding to 1,000 Hz with a level adjustment factor of 0.2 and 0.05 after the first reversal. The start values were 300 CL (T-level) and 500 CL (M-level). Moreover, the familiarization procedure served for adjustment of the stimulation parameter if required. If the listener raised the presentation level to 1,200 CL without reaching the M-level, the pulse width was increased by 10 μs. After the familiarization, the T- and M-level were measured for each of the six electrodes from apical to basal with a level adjustment factor of 0.05. For the T-level the mean result of the familiarization was used as the start value for the first electrode. Afterward the T-level of the previous electrode was used. The same procedure was used for the M-level, however, using 80% of the start value or the previous M-level to avoid overly loud stimulation. The electrical DR was calculated as the difference between the M-level and T-level for each electrode. For the U-level the start value was 90% of the electrodes M-level and the adjustment factor also was set to 0.05.

#### Categorical loudness scaling

To investigate the spectral and binaural loudness summation in the different listener groups (NH, uCI, and biCI), the ACALOS procedure (Brand and Hohmann, 2002; ISO16832, 2006) was applied for both acoustical and electrical measurements. Within the ACALOS procedure listeners judge the perceived loudness of presented signals of different levels on a scale with eleven categories from “not heard” to “too loud” covering the whole auditory dynamic range. Each of the eleven loudness categories corresponds to categorical units (CU), where 0 CU represents “not heard” and 50 CU “too loud”. The step size between the categories is 5 CU. The procedure is segmented into two phases: the individual dynamic range of hearing is estimated within the first phase and the second phase presents estimated levels in a random order to obtain loudness ratings at 5, 15, 25, 35, and 45 CU (Brand and Hohmann, 2002). The complete loudness scaling outcome for one signal consists of 22–25 loudness ratings (see Oetting et al., 2014 for more details). In the current study, the maximum presentation level was limited to the level rated with “very loud” (45 CU) instead of “too loud” (50 CU). For the electrical stimulation, a comparable method as in Theelen-van den Hoek et al. (2014) was used and accordingly, the level adjustment in the electrical domain was performed in %DR instead of dBHL. The DR of the used electrodes was estimated manually. The DR was defined between the levels that produced a “very soft” (5 CU) and a “loud – very loud” (40 CU) perception. For safety reasons the upper limit served as the maximum presentation level within the loudness scaling.

##### Monaural narrowband loudness

The ACALOS settings from Ewert and Oetting (2018) were used in the acoustical domain and transformed to the electrical domain. The initial level was 65 dBHL/65% DR (acoustic/electric), the minimal presentation level was set to –10 dBHL/–10% DR, and the level was limited to 105 dBHL in the acoustical domain. The maximum presentation level in the electrical domain was set to the maximum of the M-level and U-level from the pretests to reach the possible highest loudness range, which leads to an individual maximum presentation level of ≥ 100% DR. Each listener performed one training session in the acoustical, electrical or both domains to familiarize with the procedure and the stimuli, where the 1000 Hz narrowband noise (acoustic domain), and/or the electrode that corresponds to the center frequency of 1000 Hz (electric domain) was used. The six acoustical and/or electrical narrowband noises were measured monaurally in each ear, depending on the listener group. The stimuli were presented in a pseudo-random order. In total three repetitions of the monaural narrowband measurement were performed for each ear and each of the six frequencies or electrodes.

The responses of each run of the acoustical ACALOS procedure were fitted with the BTUX fitting method (a fitting method for loudness functions in ACALOS with threshold estimation and limitation of the uncomfortable level) as recommended in Oetting et al. (2014) and used in Ewert and Oetting (2018). Briefly, the BTUX fitting method is composed of two linear functions connected with a smoothing Bezier function (indicated as B) between 15 and 35 CU, minimizing the deviation in level-direction (indicated as X). The monotonic increasing loudness function includes a threshold estimation at 2.5 CU (indicated as T) and is limited by the uncomfortable loudness (indicated as U). If less than four responses in the upper loudness range occur, the slope of the upper loudness function is set to 1.53 CU/dB (Oetting et al., 2014). As this assumption might be invalid for CI users, the estimation of the level at uncomfortable loudness was not applied in the electrical domain (named BTX fitting method). The BTUX and BTX fitting methods also contain a stronger weighting of responses in the upper loudness region. However, pilot measurements showed a good approximation by using the BTX fitting method without any response-weighting, which allows the most flexible fitting by keeping the threshold estimation at 2.5 CU. Note that the threshold estimation in the electrical domain should correspond approximately to the predefined T-level at 0% DR. To average the three runs of the six narrowband measurements for each ear and listener, the levels corresponding to the eleven loudness categories were derived from the three fitted loudness functions and averaged for each category. Finally, the BX fitting method (no threshold estimation and no limitation of the leval at uncomfortable loudness) was used to fit the averaged loudness function to the resulting eleven mean values (Ewert and Oetting, 2018). The loudness functions of the six narrowband noises were obtained monaurally for the left and right ear, respectively.

##### Monaural and binaural equal categorical loudness noise

The same ACALOS procedure as used in Ewert and Oetting (2018) was applied to measure the loudness perception of the monaural and binaural ECLN. The ECLN consists of six narrowband noises at equal categorical loudness. To create the ECLN with a specific loudness of its components, the six narrowband noises are summed with the individual level that evoked the same loudness in CU. The equal categorical loudness of the single components is then varied within the ECLN loudness scaling procedure. To obtain the required levels of the individual narrowband components, a mapping of the narrowband CU values in a range of –5 to 50 CU to level was obtained from the narrowband loudness functions in each ear (see “Monaural narrowband loudness”). Negative CU values for the individual narrowband components were possible as the combination of six components might be audible due to loudness summation effects. Although negative CU values appear counterintuitive, they have to be understood as a mathematical concept. In fact, this is consistent with Buus et al. (1998), where loudness summation of subthreshold components in a tone complex resulted in above-threshold loudness. Because the adaptive level estimation in the ECLN procedure uses the narrowband loudness values in CU instead of the signal level, the procedure can be straightforwardly applied to the electrical domain. The measurements started with a presentation of 15 CU narrowband loudness. As in Ewert and Oetting (2018), the maximum presentation level was either limited to 50 CU narrowband loudness or to an overall level of 105 dB SPL in the acoustical domain. Based on the assumption of loudness summation effects across multiple electrodes in the electrical domain, an additional level constraint had to be found in this domain. Therefore, the CI listeners had to perform the U-level task once again by adjusting the ECLN instead of the narrowband noises to their maximum presentation level. For this, the presentation of the narrowband components of the ECLN was limited to 50 CU, starting with 10 CU and was adjusted with 3 or 1 CU steps after the first reversal. Consequently, either the obtained broadband U-level or 50 CU was used as an up-boundary in the electrical domain.

In the binaural case, the minimum of the left and right maximum presentation level was used as limitation. The listeners performed three runs of the monaural and binaural ECLN measurements. The pooled data of all repetitions were used and responses at 0 CU were excluded. Based on the analysis suggested by Ewert and Oetting (2018), the ECLN loudness function was represented by a regression line between the loudness ratings of the ECLN measurements and the narrowband CU values of the single components, minimizing the error in *x*-direction. The regression lines were used to calculate the loudness differences in CU, enabling comparison in the amount of loudness summation between the three listener groups. It should be noted that the ECLN loudness ratings include the combined effect of an increased overall level and spectral loudness summation as well as binaural loudness summation in the binaural case.

##### Binaural modified equal categorical loudness noise

The above described binaural ECLN measurement is based on the same narrowband loudness (from section “Monaural narrowband loudness”) of the individual components in each ear. The loudness of the ECLN stimulus might be unbalanced across ears, if the loudness summation is different in two ears (e.g., both electric in biCI users or acoustic and electric in uCI users). Particularly in uCI listeners, loudness summation could be expected to differ in the electric and acoustic ear. To address unbalanced loudness perception across ears in the binaural ECLN measurement, the here suggested modECLN is based on the (broadband) monaural ECLN results in each ear. To generate the modECLN at *x* CU, the fitted regression lines of the monaural ECLN results were used to determine the loudness of the six narrowband noises that result in the same required broadband loudness perception in both ears. The six narrowband noises were then presented at the level required for achieving the same broadband loudness in each ear. Any differences between the underlying narrowband loudness in the left and right ear are due to a different loudness summation in each ear. Like for the ECLN measurement, the modECLN procedure adapts the loudness values in CU instead of the signal level. The CU range was either between 0 and 50 CU broadband monaural loudness, or limited by the minimum of the U-level of the ECLN in the electrical domain and the CU values corresponding 105 dB SPL in the acoustical domain. Again, three runs of the modECLN measurement were conducted and a regression line was fitted to the CU rating of all three runs as a function of the used monaural broadband loudness. Compared to the binaural ECLN results, which include the effect of binaural loudness summation, spectral loudness summation, and the effect of an increased overall level, the modECLN results represent only the effect of binaural loudness summation.

#### Binaural loudness balancing

In the binaural loudness balancing experiment, the presentation level of one ear (reference-ear) was set to a fixed broadband loudness while the presentation level of the contralateral ear (test-ear) was self-adjusted by the listeners via responding whether the level of the left or right ear was perceived louder. The presentation level of the test-ear started with 20 CU broadband loudness and was adjusted according to the listener’s response, by 3 or 1 CU after the first reversal until the listener perceived equal loudness in both ears. As a familiarization there was one training session for each test ear with a reference level of 25 CU. Altogether four conditions were tested in the main experiment, each repeated three times: two test ears (left/right, or acoustical/electrical) and two reference levels (15, 25 CU). For each pair of loudness matched ECLNs, the respective narrowband loudness was obtained to calculate the narrowband loudness difference between the ears.

## Results

### Pretests

Within the pretests the DR of each probe electrode was calculated as the difference between the measured T- and M-level in CL. Table 2 shows the DR and the used pulse width for the 8 CI listeners. The mean DR and its standard deviation was 487 ± 185 CL. Additionally, the U-level measurement was used to obtain a higher maximum presentation level for the ACALOS procedure. To verify whether the additional task led to significantly higher level adjustments compared to the M-level results, a one-tailed dependent t-test was performed on the pooled 12 CIs (2 × 4 biCI + 4 uCI) showing a significant [*t*(11) = –2.597, *p* = 0.0125] increase in the U-levels (mean = 726 CL) compared to the M-levels (mean = 646 CL).

### Narrowband loudness functions

#### Averaged narrowband loudness functions

**FIGURE 2:**
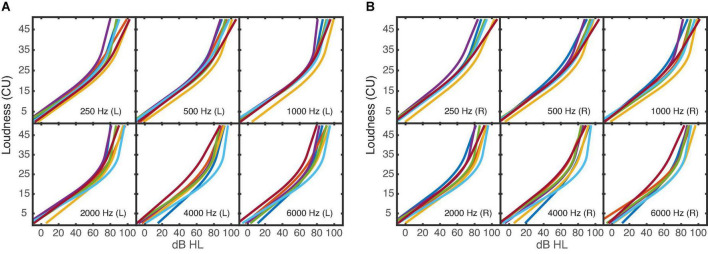
Averaged loudness functions of the six narrowband noises for each of the seven NH listeners. **(A)** Shows the results of the left ear, **(B)** of the right ear.

The six averaged narrowband loudness functions for each ear of seven NH listeners are shown in Figure 2. The results of one NH listener are excluded here and in the further tests, given that the loudness measurements did not yield enough answers in the high loudness range and it can be assumed that the fitted and averaged loudness functions did not well reflect the narrowband loudness perception of this listener^1^. Observed narrowband loudness functions for the NH listeners are well in line with results from other studies (e.g., Oetting et al., 2016; Ewert and Oetting, 2018) and show a shallow slope for low levels of about 0.32 dB/CU and an increasing slope of 1.49 dB/CU at high levels.

**FIGURE 3:**
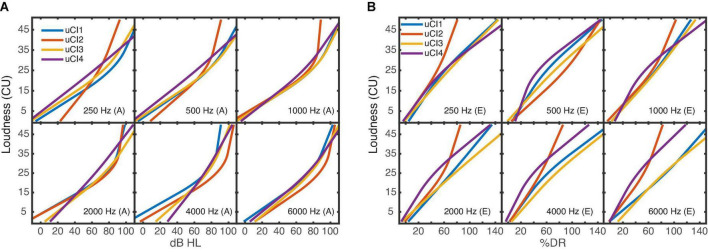
Averaged loudness functions of the six narrowband noises for each of the four uCI listeners. **(A)** Shows the acoustical measurements; **(B)** the electrical measurements of each listener. The different *x*-axes (either dBHL or% DR) depend on the used domain. The stimulations of 0% DR and 100% DR corresponded to the listeners’ T- and M-level thresholds. It should be noted that the loudness functions can have a different range across listeners and electrodes due to individual U-levels (>100% DR) which were used as maximum presentation level.

Figure 3A shows the averaged loudness functions for the uCI listeners’ NH ears in the acoustic domain. The averaged loudness functions measured in the electrical domain are shown in Figure 3B. Instead of the level in dBHL, the % DR used for stimulation is shown on the *x*-axis. The loudness functions in the acoustic ear (Figure 3A) show a larger variability than those observed in the NH group (Figure 2). Listener uCI4 shows quite straight loudness functions. In the electric ear (Figure 2B), the variability is in the same range as in the acoustic ear, however, the loudness functions deviate from the typical expansive shape with increasing slope toward higher levels as observed in the acoustic domain: in some cases, the slope decreases with increasing stimulation level (compressive behavior), otherwise the functions are more linear and a few are expansive. Linear and expansive electric loudness functions are in line with the literature (Zeng and Shannon, 1992; Theelen-van den Hoek et al., 2014). It should be noted that the slope of a loudness function depends on both the abscissa and the ordinate. Therefore, cautious should be taken when comparing the slopes between the acoustical and electrical hearing. Unexpectedly, for listener uCI2 (red line) stimulation levels beyond about 70% DR were already rated with 45 CU (“very loud”) in most cases.

**FIGURE 4:**
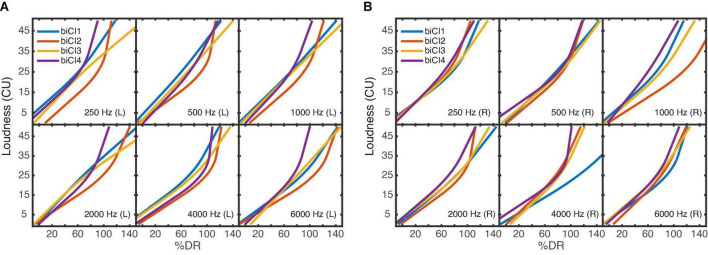
Averaged loudness functions of the six narrowband noises for each of the four biCI listeners. **(A)** left ear, **(B)** right ear. The stimulations of 0% DR and 100% DR corresponded to the listeners’ T- and M-level thresholds. As in Figure 3 the loudness functions can have a different range across listeners and electrodes due to individual U-levels.

Figure 4 shows the averaged loudness functions for the biCI listeners. Overall, the fitted loudness functions in the electrical domain show a quite individual course with larger variability across listeners than observed for NH. In contrast to the electric ear in the uCI listeners, the slope increases with increasing stimulation level as found in NH, or shows a nearly linear behavior, in agreement with the literature (Zeng and Shannon, 1992; Theelen-van den Hoek et al., 2014). The loudness functions of the right ear for the listeners biCI2 (1,000 Hz) and biCI1 (4,000 Hz) deviate most from the other loudness functions and show a considerably flatter slope. In those cases and listeners, the U-level was considerably higher than the M-level and accordingly levels up to the U-level (>100% DR) were presented during the measurement.

#### Reproducibility of the individual loudness functions

Since further measurements were based on the averaged narrowband loudness functions, it was analyzed how well the average represents the individual responses of each listener. Based on the approach of Oetting et al. (2014), the root mean square error (RMSE) between the level estimated by the averaged loudness function *F*(*x*) and the acquired data was calculated. For this, the median level $\hat{L}$ of the responses of all three runs was calculated for each loudness category *x* and the inverse loudness function *F*^−1^(*x*)was used to calculate the corresponding estimated level. Instead of all eleven loudness categories, $\hat{L}$ up to and including 45 CU were used:


$$R\,M\,S\,E\,=\,\sqrt{\frac{1}{10}\,\sum_{x=0}^{9}{\left(F^{-1}\,\left(5*x\right)-{\hat{L}}_{5*x}\right)}^{2}\,}$$

The RMSE calculations were performed for all frequencies and each ear of the NH and biCI listeners. The electrical and acoustical measurements of the uCI listeners were considered separately (see Table 3). The possible dynamic range used for the ACALOS procedure differs between the electrical (–10% DR to U-level) and acoustical domain (–10 to 105 dB SPL). For better comparability of the RMSE in % DR and dBHL, the RMSE values were scaled to the same range. For this, the range between the levels that evoked 2.5 CU and 45 CU in each narrowband measurement was calculated in both domains, leading to a mean range of 90 dBHL for the acoustical and 116% DR for the electrical domain. Hence, all electrical RMSE values were multiplied with a factor of 0.78 (= 90/116) to yield an equivalent $\tilde{\text{dBHL}}\,$ estimate. Table 3 shows the (equivalent) RMSE values and standard deviations. The equivalent RMSE values show a good agreement between the electrical measurements of the uCI listeners and the biCI listeners. Furthermore, the RMSE values of the acoustical side of uCI measurements are higher than those of the NH listeners and are more comparable to the electrical measurements. However, cautions should be taken when comparing between acoustical and electrical hearing. The comparatively higher RMSE values in the uCI and biCI users are partially because a larger level range was rated with the same loudness in some listeners, e.g., uCI2 and biCI1 did not use the full set of response options for the loudness rating.

**TABLE 3:** Root mean square error (RMSE) and standard deviation for the different listeners groups.

	NH	Acoustical uCI	Electrical uCI	biCI
Original RMSE	6.5 ± 3.5 dBHL	9.5 ± 5 dBHL	11.7 ± 8.0% DR	14.0 ± 7.4% DR
Equivalent RMSE			9.1 ± 6.2 $\tilde{\text{dBHL}}$	10.9 ± 5.7 $\tilde{\text{dBHL}}$

Additionally, the equivalent RMSE errors scaled with a dynamic range dependent factor of 0.78 in $\tilde{\text{dBHL}}$ are shown for better comparability of the electrical and acoustical domain.

#### Comparison of loudness scaling outcome with prestest T/M-levels

The electrical ACALOS procedure is based on the results of the pretests, since the level is adapted in % DR and the procedure is limited by the U-level. By using the BTX fitting method, the hearing threshold is estimated from the fitted loudness functions at the 2.5 CU (Oetting et al., 2014). In the current experimental design this value should correspond to approximately 0% DR. To examine this, the % DR at 2.5 CU was extracted for each measured electrical narrowband loudness function of each uCI and biCI listener. A total of 216 thresholds were determined and the mean threshold and standard deviation was 3.23 ± 7.89% DR. In addition, the % DR values were transformed to CL values and were compared to the T-level from the pretest. Pearson’s correlation coefficient [*r* = 0.75, *p* < 0.001] showed a highly significant correlation between the T-levels from the pretests and the hearing thresholds extracted from the loudness functions.

To assess the correlations for the pretest M-level and the loudness scaling, the mean loudness rating at a presentation of 100% DR was determined, resulting in a mean loudness of 37 CU. The level that evoked the 37 CU loudness rating was then extracted from the averaged loudness function of each listener resulting in 60 M-levels derived from the loudness functions. Spearman’s correlation analysis showed a highly significant correlation [*r* = 0.89, *p* < 0.001] between the pretest M-level and those derived from the measured loudness functions.

### Equal categorical loudness noise

**FIGURE 5:**
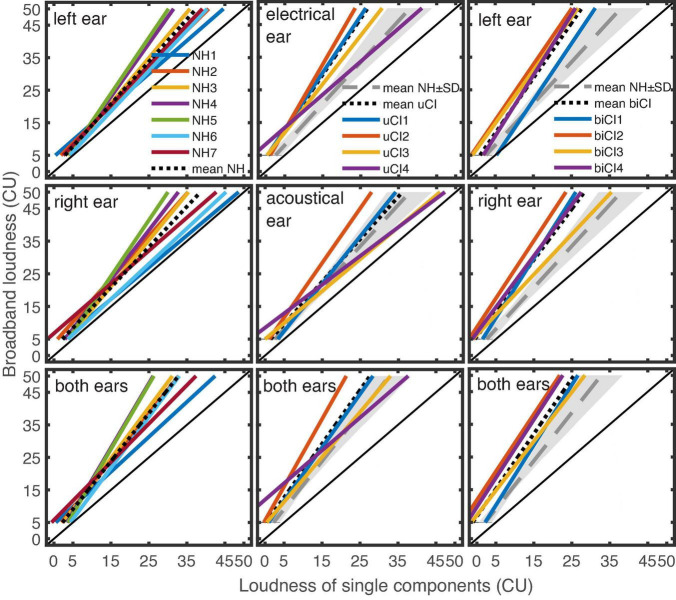
Regression lines between the loudness ratings of the monaural left, monaural right, and the binaural ECLN presentation **(top to bottom)** and the loudness of the single narrowband components for the seven NH listeners (left column), four uCI (middle column) and the four biCI listeners (right column). The dotted lines indicate the mean regression lines. The gray lines and areas indicate the averaged NH reference ± standard deviation (SD).

As described in the method section (“Monaural and binaural ECLN”), linear regression lines were fitted between the loudness ratings of the ECLN measurements and the used CU loudness of the single narrowband components. These linear regressions have been used to represent the ECLN loudness functions by Ewert and Oetting (2018) motivated by a high goodness of fit value *R*^2^ for NH and HI listeners. Here interquartile ranges of *R*^2^ between [0.92 0.94] for the NH listeners, [0.83 0.93] for the uCI listeners, and [0.84 0.89] for the biCI listeners were obtained and the regression lines were used for further analyses. Figure 5 shows the results of the monaural and binaural ECLN measurements of the seven NH (left column), four uCI (middle column), and four biCI listeners (right column) as well as the mean regression lines of each group. The mean regression lines were fitted to the averaged narrowband loudness values that evoked 5, 15, 25, 35, 45, and 50 CU ECLN loudness. The usage of CU enables the direct comparison between groups as well as between acoustical and electrical hearing. Since further analysis generally does not distinguish between left and right but between acoustical and electrical, an average monaural reference was derived from the regression lines of both ears of the seven NH listeners. For the uCI and biCI listeners, additionally, these average regression lines and corresponding standard deviations are also plotted as NH monaural and binaural references (long dashed line and gray area).

Higher loudness ratings for the ECLN stimulus compared to the narrowband loudness results in a vertical offset of the regression lines to the diagonal line and can be explained by spectral and binaural loudness summation in addition to the increased overall level. Figure 5 shows that all listeners perceived an increased ECLN loudness compared to the narrowband loudness of the single components and the binaural ECLN ratings were higher than the monaural ECLN ratings.

Regarding the difference between CI and NH listeners, any offset to the left-up direction of the regression lines from the NH reference line (long dashed), indicates an increased spectral loudness summation in the monaural measurements and an increase in the combination of spectral and binaural loudness summation in the binaural measurements compared to NH listeners. Overall, Figure 5 shows that the electrical ECLN loudness was rated higher by most biCI and uCI listeners compared to the NH loudness rating.

**FIGURE 6:**
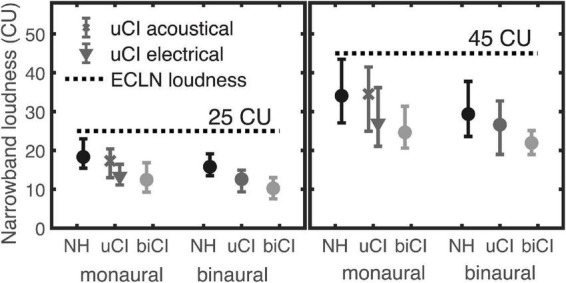
Narrowband loudness that evoked a 25CU (left panel) and 45CU (right panel) loudness rating of the ECLN for the different listener groups. Values were extracted from the regression lines of each listener and the error bars show mean and range of the data.

To analyze the difference between the three listener groups, the narrowband CU values that evoked responses of 25 and 45 CU for the ECLN were extracted from the monaural and binaural linear regressions. Monaural left and right data were pooled for NH and biCI listeners, whereas the electrical and acoustical measurements of the uCI listeners were analyzed separately. Figure 6 shows the mean and the range of the narrowband loudness that evoked 25 CU (left panel) and 45 CU ECLN loudness (right panel) in the different listener groups. The horizontal dashed lines represent the same narrowband and ECLN loudness. Obviously, the biCI listeners required lower narrowband loudness of the single components of the ECLN compared to the NH listeners in all conditions. Furthermore, monaural results at 25 CU loudness rating of the uCI listeners show that the mean values of the acoustical measurement (indicated by the *x* symbol) almost correspond to those of the NH group (17.3 and 18.32 CU); likewise the mean values of the electrical measurements almost correspond to the biCI results (13.32 and 12.45 CU). The same effect can be observed in the narrowband loudness that evoked 45 CU monaural ECLN loudness. In both cases the mean narrowband loudness in the binaural case of the uCI listeners is centered between the results of the NH and biCI listeners. The range of the data is considerably larger at 45 CU than at 25 CU.

Two-way repeated-measures ANOVA (ear × ECLN level) showed a significant difference between the acoustical and electrical ear in uCI listeners (*p* < 0.01), however, there was no significant main effect of ear (left and right) for both NH and biCI listeners. Thus, in Figure 6 and later analyses, the mean values of the left and right ear were used in the NH and biCI groups. A mixed-design ANOVA (between subjects factor listener group × within-subjects factor ECLN level) showed a significant effect of level (*p* < 0.001). There was no significant difference between the acoustic hearing of uCI and NH listeners, as well as between the electrical hearing of the uCI and the biCI users. A mixed-design ANOVA (between subjects factor NH and biCI × within-subjects factor ECLN level) showed significant main effect of level (*p* < 0.001) and group (*p* < 0.01), indicating a significantly larger monaural loudness summation in the electrical hearing than in the acoustic hearing. For the binaural cases, a mixed-design ANOVA (between subjects factor NH, uCI, and biCI × within-subjects factor ECLN level) showed significant effect of level (*p* < 0.001) and group (*p* < 0.05).

To determine the necessary number of participants, a sample size analysis was performed with G*Power 3.1 (Faul et al., 2009). In general, as expected, most test conditions require a larger sample size, even thought the effect size in this study was considered to be very large using Cohen’s (1988) criteria. For example, the effect size calculated based on the pooled acoustical ear results of NH and uCI listeners (mean 18.09, standard deviation 2.51) and pooled electrical ear of uCI and biCI listeners (mean 12.73, standard deviation 2.36), for the 25 CU shown in Figure 6, is 2.22. For an alpha = 0.05 and power = 0.80, the projected sample size is approximately *n* = 4 subjects per group. For comparing the bilateral loudness summation between groups, i.e., NH vs. uCI, uCI vs. biCI, and biCI vs. NH listeners, the effect size/project sample size is 1.49/7, 0.944/15, and 2.44/4, respectively at 25 CU.

### Modified equal categorical loudness noise

**FIGURE 7:**
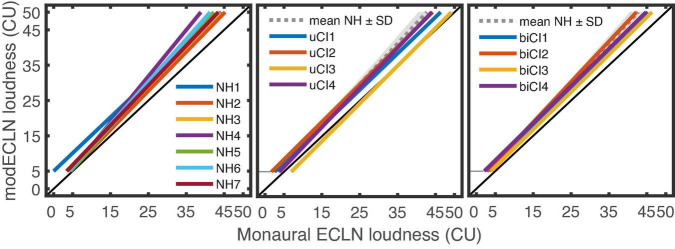
Regression lines between the loudness ratings of the binaural modECLN presentation and the loudness of the monaural ECLN for the seven NH, four uCI and four biCI listeners. The gray line and areas indicate the averaged NH reference ± one standard deviation.

As for the ECLN measurements, linear regression lines were fitted between the loudness ratings of the modECLN measurements and the used monaural ECLN loudness. Here interquartile ranges *R*^2^ were between [0.89 0.94] for the NH listeners, [0.85 0.92] for the uCI listeners, [0.82 0.88] for the biCI listeners. The results for the NH, uCI, and biCI listeners are shown in Figure 7 and again the NH reference (dashed line) is shown for group comparison. In this case a vertical offset of the regression lines compared to the diagonal line indicates binaural loudness summation, i.e., a higher loudness of the binaural modECLN compared to the monaural ECLN. It appears that this offset is approximately comparable among the three listener groups.

**FIGURE 8:**
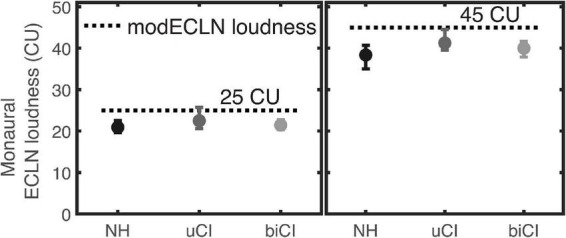
Monaural ECLN loudness that evoked 25CU (left panel) and 45CU (right panel) loudness rating of the binaural modECLN for the different listener groups. Values were extracted from the regression lines of each listener and the error bars show mean and range of the data.

For a better group comparison, the monaural ECLN loudness was extracted that evoked 25 CU and 45 CU loudness ratings of the binaural modECLN for the different listener groups. Results are shown in the left and right panel of Figure 8. Differences between the listener groups indicate differences in the amount of binaural loudness summation. The groups show similar results, indicating only small differences in the amount of binaural loudness summation. For both loudness ratings there is a slight tendency of higher required monaural ECLN values for the uCI listeners compared to the NH and biCI listeners. The highest difference of 2.9 CU can be determined between the uCI and the NH listeners in the 45 CU binaural modECLN case. However, a mixed-design ANOVA (between subjects factor NH, uCI, and biCI × within-subjects factor modECLN level) showed no significant difference between groups.

### Binaural loudness balancing

Binaural loudness balancing was performed to cross validate how well the modECLN, assuming equal monaural broadband loudness in each ear, matches the self-adjusted loudness in each ear, required for a balanced broadband loudness perception across both ears. The experiment determined the loudness difference between the left and right ear that was required to match the broadband loudness in both ears for a 15 and 25 CU reference. In order to directly compare the self-adjusted between-ear differences with the corresponding differences as assumed in modECLN based on the monaural ECLN, the obtained self-adjusted monaural broadband loudness at the test-ear as well as the reference loudness at the contralateral ear was transformed to the underlying narrowband loudness of the single components using the ECLN regression lines. For difference calculation, the right loudness values were subtracted from the left ones for NH and biCI. For the uCI listeners the electrical loudness values were subtracted from the acoustical ones. The calculated narrowband loudness differences of the three repetitions at each adjusted-ear side were averaged, resulting in one narrowband loudness difference for each listener and each tested level. For modECLN, the individual narrowband loudness differences between ears were calculated in the same way.

It was found that two uCI listeners had difficulties to adjust the loudness of the electrical ear to that of the acoustical ear, given that the electrical signal was always perceived louder. This suggests that the minimum perceived loudness in the electric ear starts at certain above-threshold loudness or that the loudness sensation is accompanied by a non-auditory sensation adding a cross-modal component to the balancing task. For this reason, three data points of listener uCI2 and two data points of listener uCI3 were excluded for the analysis of 15 CU ECLN loudness.

**FIGURE 9:**
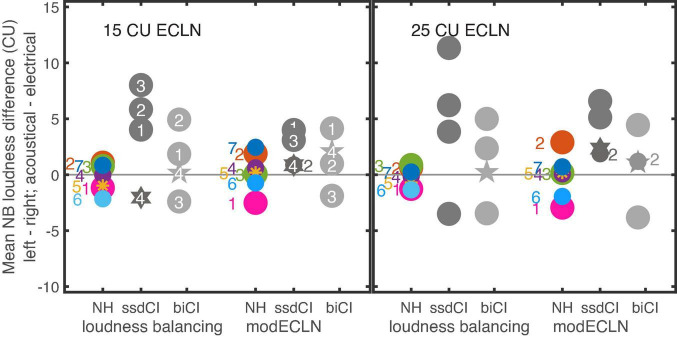
Mean narrowband (NB) loudness difference that evoked 15CU (left panel) and 25CU (right panel) ECLN loudness on both ears. Right side of each panel shows the difference that is estimated by the modECLN procedure and left side shows the self-adjusted difference for the NH, uCI and biCI listeners. The numbers represent the individual listeners. The modECLN calculated nearly same NB differences for listener uCI2 and uCI4 at 15CU and listener biCI2 and biCI4 at 25CU ECLN loudness.

Figure 9 shows the mean narrowband loudness differences derived from the balancing experiment for the NH, uCI, and biCI listeners for the two reference loudness values. Additionally, the corresponding narrowband loudness differences as assumed in modECLN are shown for each listener. Positive values represent higher required narrowband loudness at the left (NH, biCI) or acoustical (uCI) ear to evoke equal broadband loudness in both ears. At both levels for the NH listeners narrowband loudness differences extracted from loudness balancing are close to 0 CU, while the modECLN stimulus predicted differences in a slightly higher range, resulting in differences around ± 2 CU. Loudness balancing results of the biCI listeners still cluster around 0 CU, but show more deviations compared to the NH listeners. The narrowband loudness differences for the modECLN are in the same range. Especially for 25 CU the order of the biCI listeners is comparable, resulting in very small differences between the results from binaural loudness balancing and modECLN. In this case the narrowband difference estimated by the modECLN deviates with ± 1.2 CU from the loudness balancing outcome of the biCI listeners. Listener biCI2 showed a deviation in the case of 15 CU, as the required narrowband loudness difference was underestimated by 4 CU for the modECLN.

Highest narrowband loudness differences for loudness balancing up to 11 CU were found for the uCI listeners. Here the narrowband loudness used in the modECLN was higher for the acoustical ear side for all uCI listeners. The same tendency is observed for listeners uCI2 and uCI3 for the loudness balancing, however, with considerably higher differences. For uCI1 the narrowband loudness difference for balancing and modECLN is the same (4 CU) in the 15 CU case and differs about 1 CU in the 25 CU case. In contrast to modECLN, the balancing results for listener uCI4 showed a higher loudness of the individual components in the electrical ear.

## Discussion

### Loudness summation of equally loud narrowband signals

In the current study, the listeners of all groups showed loudness summation (including the effect of an increased overall intensity), given that less than 25 CU narrowband loudness of the single components was required to evoke 25 CU monaural ECLN loudness. The same effect could be shown for a monaural ECLN loudness of 45 CU. Furthermore, the effect of loudness summation increases with increasing ECLN loudness for all three listener groups. The results for the NH and HI listeners (Ewert and Oetting, 2018) indicate an increasing effect of loudness summation. However, no significant difference between 25 and 45 CU could be shown after a transformation to the interval scale in phon (Ewert and Oetting, 2018). Theelen-van den Hoek et al. (2015) reported a decrease in the amount of spectral loudness summation for the loudness categories “very soft” and “soft” compared to the categories up to “medium – loud.”

The results of the present study showed a higher monaural loudness summation for the four biCI listeners compared to the NH group. With a median narrowband loudness of 12 and 23.5 CU for evoking 25 and 45 CU monaural ECLN loudness, respectively (Figure 6), which show a higher effect of loudness summation in comparison to the HI listeners (17.2 and 31.8 CU) in Ewert and Oetting (2018). The results of the uCI listeners show that the loudness summation of equally loud narrowband noises in the acoustical ear corresponds to that of the NH listeners and in the implanted ear to that of the CI listeners. These results confirm the expectations of unbalanced loudness perception for all four uCI listeners (Figure 5). Accordingly loudness perception of the four uCI users with NH in the contralateral ear appears to be strongly dominated by the electrical ear. Since the effect of loudness summation in the electrical domain is also higher than in the HI group, an unbalanced loudness is likely to also occur for bimodal listeners with a hearing aid at the contralateral ear. However more data are needed to draw stronger conclusions. The current NH results can be directly compared to the according data in Ewert and Oetting (2018): the monaural results for 25 and 45 CU ECLN show median values of 17.9 and 33.6 CU narrowband loudness, and are slightly lower than the obtained median values of 19.9 and 36.5 CU narrowband loudness in Ewert and Oetting (2018). The same effect is observed for the binaural measurements. These differences are comparatively small and approximately in the range of standard deviations, and might be caused by the limited sample size of the groups of seven and nine NH listeners in both studies. A further potential reason for the differences between the studies might be the adjustment of the measurement procedure: here the maximum presentation level was limited to 45 CU instead of 50 CU in Ewert and Oetting (2018). Accordingly, the range of the presented levels slightly changed and the loudness ratings of the listeners might have changed as an effect of the so called “stimulus range equalizing bias” (Poulton, 1989).

Classical experiments to investigate spectral loudness summation in the acoustical domain increase the bandwidth of the signal while keeping the intensity constant. Theelen-van den Hoek et al. (2015) designed an analogous measurement procedure for CI users assuming that the increasing bandwidth corresponds to multi-electrode stimulation in the electrical domain. Constant intensity was achieved by using the same overall stimulation rate. In order to avoid the effect of different DRs between the electrodes, a similar approach to that of the ECLN was used, i.e., the averaged level in μA of all single electrodes with the same loudness was used for the loudness summation calculations. However, since the stimulation rate of the individual electrodes was reduced for the multi-electrode stimulation, it should be noted that the same loudness of individual electrodes was not necessarily provided at reduced stimulation rates. This assumption is only valid if the loudness functions of the individual electrodes change equally with different stimulation rates. Nevertheless, additional analysis performed in Theelen-van den Hoek et al. (2015) indicated a similar effect of changes in stimulation rate on loudness perception for different electrodes and electrode combinations. The ECLN sums six narrowband noises with the same loudness. In contrast to the classical spectral loudness summation experiments at constant intensity, the ECLN results in an increase of overall intensity in the acoustic domain, and accordingly in an increase of the overall stimulation rate in the electrical domain. Thus, the ECLN results contain both effects on the listeners’ loudness perception, the effect of increased overall intensity or overall stimulation rate and the effect of spectral loudness summation. It can be assumed that most of the observed loudness increase can be explained by spectral loudness summation in the electrical domain, given that Theelen-van den Hoek et al. (2015) reported that the effect of spectral loudness summation increased with increasing loudness categories up to “medium” (25 CU) relative to the effect of stimulation rate. For a comparison of the results of acoustic and electric domains it remains, however, unclear if the effect of the increased intensity or stimulation rate is comparable in both domains.

The analysis in the present study investigated the amount of loudness summation of equally loud signals and does not allow drawing conclusions about potential differences between the underlying mechanisms in the acoustical domain and the electrical domain, such as the amount of loudness summation caused by channel interactions (e.g., spread of electric charge). Further research including loudness models would be necessary to investigate these differences and to compare effects of spread of excitation on the basilar membrane of NH and channel interactions in CI. Independent of the mechanism contributing to increased loudness summation in the electrical domain, unbalanced loudness perception should be considered in clinical fitting of unilateral CI users with a normal hearing (uCI) or aided hearing impaired contralateral ear (bimodal CI). The current results suggest that a balanced loudness between narrowband signals and the equivalent single electrode stimuli is not sufficient for a balanced loudness perception, and more complex stimuli should be taken into account given that broadband signals occur primarily in everyday situations.

### Binaural loudness summation

For the uCI group, the binaural ECLN measurements were similar to the monaural electrical measurements, indicating a dominating electrical ear in the binaural measurement and little contribution of the softer acoustical ear to binaural loudness summation. In contrast, Blamey et al. (2000) observed binaural loudness summation in bimodal CI listeners, especially for equally loud acoustical and electrical components on both ears. For the modECLN, which aims to present the same loudness in both ears (see discussion below) binaural loudness summation was observed for the uCI listeners. However, the amount of binaural loudness summation appeared to be slightly reduced compared to the NH and biCI listeners. Binaural loudness summation in NH and biCI listeners was comparable for ECLN and modECLN, indicating that there was no dominance of one ear and thus more similar loudness summation in both acoustic and electric ears.

Ewert and Oetting (2018) showed an increased binaural loudness summation for a subset of the tested HI listeners. Such an increased binaural loudness summation has neither been demonstrated for the current biCI nor for the subset of uCI listeners when compared to the NH group. However, such an increased binaural loudness summation cannot be excluded for biCI and uCI listeners, given the small sample size of four listeners in each group. The mechanism behind such an increased binaural loudness summation has not been investigated yet and further research is required to assess a similar potential effect in the electrical domain. For this, the equally loud signals employed here appear to be suitable, offering a good comparability between different listener groups.

### Relation between fitting map, pretests, and loudness scaling

As the determination of the T- and M-levels represent the essential part of the CI fitting, Theelen-van den Hoek et al. (2014) examined the correlation between these fitting parameters in the CI users’ individual maps and their loudness scaling outcome as a part of their reliability tests of loudness scaling in the electrical domain. They found no significant correlation for the T-levels (*r* = 0.41) but a strong and significant correlation for the M-levels (*r* = 0.85). For these calculations they only used 15 electrodes, which were stimulated with the same pulse rate and pulse width as defined in the listeners’ fitting maps. A similar analysis was performed in the current study and the influence of the pulse width was minimized by determining the T- and M-level in electrical charge ($\frac{a\,m\,p\,l\,i\,t\,u\,d\,e\,\left(C\,L\right)\cdot p\,u\,l\,s\,e\,w\,i\,d\,t\,h\,\left(\mu \,s\right)}{1000})$ as in Racey and Dillon (2017). The current results also show no correlation for T-levels and a highly significant correlation for M-levels [*r* = 0.84, *p* < 0.001] between the values extracted from the loudness functions and those calculated from the listeners’ clinical fitting map. Furthermore, based on the current results, the M-level in the loudness scaling function was defined slightly different as the level that evoked 37 CU (averaged loudness at 100% DR) instead of 40 CU in Theelen-van den Hoek et al. (2014). It should be noted that amplitude modulated pulse trains were used in the present study, which might have caused overall higher obtained M-levels as in Theelen-van den Hoek et al. (2014). The non-significant correlation of the T-levels from the loudness scaling and the T-levels specified in the listeners’ maps in both Theelen-van den Hoek et al. (2014) and the current study could be related to a different stimulation rate (Galvin and Fu, 2005) of the stimuli used here and in the clinical fitting, as well as to potential variations of T-level in time (Theelen-van den Hoek et al., 2014). However, a significant correlation between the T-levels in the pretests and the hearing thresholds extracted from the loudness functions was found (*r* = 0.75). In this case, both measurements used the same stimulation rate and were performed within a week.

The current M-level and additional U-level measurements were used to obtain the highest possible stimulation level without an unpleasant sensation for the single electrode stimulations. The averaged maximum rating within all single electrode loudness scaling results was 42 CU. However, the usage of both, M- and U-level, produces a large spread of the % DR that was used within each narrowband measurement. Consequently, a comparison of the individual loudness functions is difficult, even though this was not the goal of the present study. Theelen-van den Hoek et al. (2014) as well as Busby and Au (2017) used the scale of the loudness scaling and defined the DR as the difference between fixed categories of the chosen scale. Their method enables a better comparability of the fitted loudness functions, because the same scale was used in the pretests and the following loudness scaling experiments. This approach additionally familiarizes the listeners with the scale. Obviously their procedure requires a further check for unpleasant sensations given that it might be not possible to stimulate the electrodes at a “very loud” perception at all (e.g., Busby and Au, 2017). Furthermore, using loudness scaling seems to be faster and therefore should be considered as the pretest to obtain the individual DRs for further investigations.

### Applicability of the binaural and modified equal categorical loudness noise measurement

The uCI results for the monaural ECLN measurements suggests a unbalanced bilateral loudness perception for the current four listeners given that the electrical ear required a lower narrowband loudness of the single components compared to the NH ear to evoke 25 CU and 45 CU broadband loudness. This finding is supported by the loudness balancing experiment where three of four uCI listeners required higher narrowband loudness in the acoustical ear to obtain the same broadband loudness as in the electrical ear. Buss et al. (2018) suggested that CI users with normal to near-normal hearing in the contralateral ear may be resistant to optimal electric stimulation levels due to the novelty and quality of the sound compared to their NH hearing ear. Binaural loudness summation is reported to be largest for equal loud signals in the acoustical (Zwicker and Zwicker, 1991; Moore and Glasberg, 2007; Moore et al., 2014) and the electrical domain (Blamey et al., 2000). Therefore, these results suggest a limited usability of the (original) binaural ECLN in uCI due to the potentially increased risk of unbalanced loudness perception in both ears.

The here suggested modECLN aims to achieve equal broadband loudness in both ears using the monaural ECLN loudness functions at equal loudness for signal generation. The loudness balancing experiment showed that the difference of the narrowband loudness between both ears is slightly overestimated by the modECLN procedure compared to the self-adjusted differences obtained from loudness balancing. However, the modECLN predicted comparable level differences as adjusted by the listeners in the 25 CU ECLN condition of the loudness balancing experiment. Thus the modECLN appears generally suited to assess binaural loudness summation ensuring a sufficiently balanced loudness across ears. Additional informal fusion tests also showed that modECLN leads to improved balancing as most listeners perceived more symmetrically distributed auditory images across ears instead of the highly dominating electrical ear in case of broadband stimuli composed of equally loud components as presented in the (original) binaural ECLN measurement. Nevertheless, almost no uCI listener perceived a single fused auditory image. However, a detailed assessment of fusion was not in the scope of the current study.

The use of a loudness-based stimulation as a comparable measure in the acoustical and electrical domain is likely restricted near the hearing threshold and the discomfort threshold. On the one hand, listeners might feel the stimulation near threshold before perceiving a sound for electrical stimulation, and on the other hand unpleasant non-acoustical sensations might occur (e.g., facial nerve stimulation) before perceiving very loud sounds for electrical stimulation. Such cross-modal sensations and comparisons could have led to the difficulties of the listeners in the loudness balancing task, particularly at the higher reference loudness (25 CU). The usage of monaural loudness functions in the modECLN has the advantage of avoiding potential cross-model comparisons. Nevertheless, cross-model sensations might be unavoidable in the underlying monaural tasks with electric stimulation, too.

Taken together, monaural loudness functions offer a good approach to provide equally loud signals in both ears, given that equal loudness of the test signals in both ears is essential for the measurement of the binaural loudness summation in unilateral CI users with residual NH in the contralateral ear. Moreover, the here suggested method is applicable in HI listeners as well, offering the opportunity to derive unified guidelines for inclusion of broadband binaural loudness-based procedures for hearing instrument fitting. In general, the usage of CU as universal unit enables direct comparison between listener groups as well as between acoustical and electrical hearing. However, it should be noted that the different ordinates in acoustical and electrical hearing should be considered when directly comparing the individual shape of loudness functions. Therefore, we suggest such comparisons should be viewed cautiously, even though this was not the goal of the present study and loudness functions served as an intermediate step for ECLN and modECLN. One limitation of the current study is the small sample size of the two CI groups. We also noted that the NH and CI listeners had some differences in age ranges. Therefore, there are potential effects of age that were not controlled for in this study.

In general, the effect size in this study was considered to be very large using Cohen’s (1988) criteria. However, as expected, the tested sample size is still too small for drawing solid conclusions on possible bilateral loudness summation differences among all three groups. Nevertheless, it appears just adequate for comparisons between NH and biCI listeners. Future research should aim to better understand unbalanced loudness summation on electric and acoustic hearing in a larger listener groups. The goal of an optimized binaural/bilateral fitting would be balanced loudness growth (thus also considering the shape of the loudness function) both for narrowband as well as broadband stimuli in both ears, independent of the mode of stimulation.

## Conclusion

The present study aimed at investigating loudness summation of equally loud narrowband signals in NH, uCI and biCI listeners within a common framework. For this, the acoustical ECLN procedure (Ewert and Oetting, 2018) was adapted to an equivalent procedure in the electrical domain using direct stimulation as well as to a combined acoustical and (direct) electrical stimulation. The developed test procedure enables categorical loudness scaling in the electrical and the acoustical domain, and simultaneous stimulation in both domains. To cope with differences in loudness summation in each ear, a modified procedure (modECLN) was introduced and evaluated, ensuring the same broadband loudness in both ears. The following conclusions about loudness summation and the employed test procedure can be drawn:

(1) Higher (monaural) loudness summation of equally loud narrowband signals was found in the electrical domain in uCI and biCI listeners compared to NH listeners. This increased loudness summation is even higher than that found in HI listeners in Ewert and Oetting (2018). In contrast to an increased binaural loudness summation observed for HI listeners in Ewert and Oetting (2018), a similar amount of binaural loudness summation was found for the current uCI, biCI, and NH listeners.

(2) The original binaural ECLN measurement appears unsuited for the current four uCI listeners due to unbalanced loudness perception in electrical and acoustical domains. The suggested modECLN provided balanced binaural loudness. The unbalanced loudness perception with the original ECLN in the current uCI listeners emphasizes the importance of including broadband measurements in the clinical CI fitting at least for loudness perception verification. A loudness balancing between narrowband signals and single electrodes does not automatically result in a balanced loudness perception between both ears.

(3) The proposed procedure might be integrated in future bilateral loudness fitting and evaluation within a common framework for electric, acoustic or electric/acoustic hearing. However, further studies with a larger CI subject pool are required before clinical application.

## Data availability statement

The original contributions presented in the study are included in the article/supplementary material, further inquiries can be directed to the corresponding author/s. The AFC and ACALOS software are publicly available at www.aforcedchoice.com.

## Ethics statement

The study was reviewed and approved by the Ethics Committee of the University of Oldenburg. All listeners provided voluntary written informed consent to participate in the study.

## Author contributions

HH and SE conceived the presented idea and supervised the project. HH and LH developed the test software. LH carried out the experiments. All authors discussed the results and contributed to the manuscript.

## Footnotes

1 For this listener, the fitting of the loudness function used approximations inherent to the BTUX procedure (Oetting et al., 2016; Ewert and Oetting, 2018) and the slope of the upper linear part of the loudness function was either set to the maximum value of 5 CU/dB (42% of the cases) or was automatically set to 1.53 CU/dB (36% of the cases) due to too few answers in the high loudness range. Uncertainties in this underlying first measurement would propagate into all ECLN measurements.

## Abbreviations

ACALOS: adaptive categorical loudness scaling
biCI: bilateral CI
BTUX: fitting method for loudness functions in ACALOS with threshold estimation and limitation of the uncomfortable loudness level
BTX: fitting method for loudness functions in ACALOS with threshold estimation
BX fitting: method for loudness functions in ACALOS
CI: cochlear implant
CU: categorical units CL current levels
M-level: maximum comfortable level
DR: dynamic range
ECLN: equal categorical loudness noise
HI: hearing impaired
modECLN: modified ECLN
NH: normal hearing
PTA4: pure-tone average over 500 1000, 2000 and 4000 Hz
RMSE: root mean square error
T-level: hearing threshold level
U-level: uncomfortable level
uCI: unilateral CI users with normal hearing in the unaided ear (single-sided deafness).
