# Corrigendum: Spectral and binaural loudness summation of equally loud narrowband signals in single-sided-deafness and bilateral cochlear implant users

**DOI:** 10.3389/fnins.2022.1087671

**Published:** 2023-01-13

**Authors:** Hongmei Hu, Laura Hartog, Birger Kollmeier, Stephan D. Ewert

**Affiliations:** ^1^Medizinische Physik and Cluster of Excellence “Hearing4all”, Department of Medical Physics and Acoustics, Universität Oldenburg, Oldenburg, Germany; ^2^Hörzentrum Oldenburg gGmbH, Oldenburg, Germany

**Keywords:** spectral loudness summation, binaural loudness summation, categorical loudness, single-sided-deafness, cochlear implant, bilateral cochlear implant fitting

In the published article, there was a mistake in the email address of the correspondence. The email address of the corresponding author is hongmei.hul@uol.de. The correct email address should be hongmei.hu@uol.de.

